# Are Twin Pregnancies Complicated by Weight Discordance or Fetal Growth Restriction at Higher Risk of Preeclampsia?

**DOI:** 10.3390/jcm9103276

**Published:** 2020-10-13

**Authors:** Veronica Giorgione, Amar Bhide, Rohan Bhate, Keith Reed, Asma Khalil

**Affiliations:** 1 Twins Trust Centre for Research and Clinical Excellence, St George’s University Hospitals NHS Foundation Trust, London SW17 0RE, UK; vgiorgio@sgul.ac.uk (V.G.); abhide@sgul.ac.uk (A.B.); m1700399@sgul.ac.uk (R.B.); 2 Vascular Biology Research Centre, Molecular and Clinical Sciences Research Institute, St George’s University of London, London SW17 0RE, UK; 3 Fetal Medicine Unit, St George’s University Hospitals NHS Foundation Trust, London SW17 0RE, UK; 4 Twins Trust, The Manor House, Aldershot GU12 4JU, UK; keithreed@twintrust.org

**Keywords:** preeclampsia, hypertensive disorders of pregnancy, twin pregnancy, fetal growth restriction, small for gestational age, birthweight discordance

## Abstract

Studies have reported controversial findings on the association between fetal growth restriction (FGR) or intertwin weight discordance and the risk of hypertensive disorders of pregnancy (HDP) in twin pregnancies. The aim of this study was to investigate the association between twin growth disorders and HDP. Twin pregnancies resulting in two live births at St George’s Hospital between 2000 and 2019 were included. FGR or small-for-gestational-age (SGA) at birth was assessed using singleton and twin reference charts. Intertwin discordance [(large birthweight − small birthweight)/(large birthweight) × 100%)] was calculated. Logistic regression models were performed. SGA (aOR 2.34, 95% CI 1.60–3.44, *p* < 0.001), intertwin discordance ≥25% (aOR 2.10, 95% CI 1.26–3.49, *p* = 0.004) and their co-existence (aOR 2.03, 95% CI 1.16–3.54, *p* = 0.013) were significantly associated with HDP. After adjusting for the known maternal risk factors of HDP and the intertwin discordance, SGA (using the twin charts) was the strongest independent risk factor associated with HDP (aOR 2.12, 95% CI 1.40–3.22, *p* < 0.001) and preeclampsia (aOR 2.34, 95% CI 1.45–3.76, *p* < 0.001). This study highlights that the presence of at least one SGA twin is significantly associated with HDP during pregnancy. Therefore, maternal blood pressure should be closely monitored in twin pregnancies complicated by SGA with or without intertwin discordance.

## 1. Introduction

The rising age at childbirth and the extensive use of fertility enhancing treatments have contributed to the increased incidence of twin pregnancies [1]. Many pregnancy complications are more commonly encountered in multiple pregnancies than in singletons, including hypertensive disorders of pregnancies (HDP), the incidence of which is at least two times higher, in both dichorionic and monochorionic twin pregnancies [2,3]. A common fetal complication of twin pregnancies is small for gestational age (SGA), defined as birthweight <10th percentile [4]. Even in the absence of SGA, twin pregnancies can be complicated by large intertwin birthweight discordance. Although the cause and pathophysiology of increased intertwin discordance are not fully understood, this condition is associated with an increased risk of adverse perinatal mortality and morbidity [5,6].

Whereas the association between fetal growth anomalies and HDP has been well established in singleton pregnancies [7], several studies investigating this relationship in twin pregnancies have reported conflicting results [8,9,10,11,12,13]. Despite previous negative findings [10,11], Proctor et al. demonstrated that the association between SGA and HDP in dichorionic twin pregnancies seems to be similar to that observed in singleton pregnancies when twin-specific birthweight references were used [8]. Moreover, a recent report has shown that discordant twins had a higher risk of preeclampsia, with a positive correlation with the degree of intertwin weight discordance [9].

We are yet to ascertain whether twin pregnancies complicated by fetal growth abnormalities are at increased risk of HDP or preeclampsia, and whether any such association differs according to chorionicity.

## 2. Experimental Section

### 2.1. Study Population

This was a cohort study with retrospective analysis of prospectively collected data. The inclusion criteria were unselected twin pregnancies with two live births at St George’s Hospital, London, UK, between January 2000 and January 2019. Pregnancies were identified by searching the electronic maternity records (ViewPoint version 5.6.26.148, ViewPoint Bildverarbeitung GMBH, Wessling, Germany). Exclusion criteria were major fetal structural anomalies or aneuploidy, twin-to-twin transfusion syndrome (TTTS), twin reversed arterial perfusion sequence (TRAPS), twin anemia-polycythemia sequence (TAPS), missing gestational age at delivery, birthweight or HDP data, and women with pre-pregnancy hypertension. Pregnancy outcomes were ascertained from the maternity database and neonatal records.

### 2.2. Study Variables and Outcomes

Data on maternal age in years, weight in kilograms, body mass index (BMI) in kg/m^2^, reported ethnicity, mode of conception and smoking were collected. The chorionicity was determined based on the number of placentas and the presence or absence of the lambda sign at the intertwin membrane–placenta junction, as well as the intertwin membrane thickness at the site of its insertion in the chorion at 11–14 weeks, or the number of placentas and the fetal gender after 14 weeks’ gestation [14,15,16]. Gestational age (GA) was determined according to the crown-rump length (in the first trimester) or head circumference (after 14 weeks’ gestation) of the larger fetus in cases of spontaneous conception and according to the timing of in vitro fertilization for pregnancies conceived via assisted reproductive technology [14,17,18,19].

Intertwin birthweight discordance was calculated using the following formula: [(larger twin Birthweight − smaller twin Birthweight)/(larger twin Birthweight) × 100%)]. Three different cut-offs (≥15%, ≥20% and ≥25%) were used to define a significant intertwin birthweight discordance [6]. SGA was defined as birthweight centile below the 10th centile and fetal growth restriction (FGR) was defined as birthweight centile of one of the fetuses below the 3th centile [3]. Although the recent Delphi consensus statement did not propose a definition for selective fetal growth restriction (sFGR) at birth, we adapted the antenatal criteria of one of the following: (1) one twin below the 10th birthweight centile and a birthweight discordance of more than 25% or (2) the solitary criterion of one twin with FGR [20]. The birthweight centiles were assessed using the singleton standard reported by Poon et al. and twin chorionicity specific standards reported by Ananth et al. [16,21].

HDP included gestational hypertension and preeclampsia, as defined by the International Society for the Study of Hypertension in Pregnancy (ISSHP) guideline [22]. Women with new-onset hypertension (≥140mm/Hg systolic or ≥90mm/Hg diastolic on two separate occasions 12 h apart) were classified as gestational hypertension or preeclampsia according to the presence of systemic involvement (significant proteinuria or maternal organ dysfunction). Significant proteinuria was defined as protein/creatinine ratio (PCR) ≥30mg/mmol. Other criteria of systemic involvement included liver dysfunction (aspartate aminotransferase or alanine aminotransferase concentrations more than twice the upper reference range), reduced platelet count (<100,000/μL), hemolysis (increased lactate dehydrogenase concentration more than twice the upper reference range), renal failure (creatinine >90 umol/L), or neurological symptoms (persistent severe headache, seizures).

### 2.3. Statistical Analysis

Categorical data were presented as number (%) and compared using the Fisher’s exact test or Chi-square test. Continuous data were presented as median (interquartile range, IQR). The D’Agostino and Pearson Omnibus test was used to assess the normality of the data. Non-parametric analysis using Mann-Whitney U-test was then used to compare continuous data between the study groups. We planned a priori sensitivity analysis according to chorionicity (dichorionic and monochorionic). Logistic regression analyses were used to assess the association between birthweight discordance, FGR or SGA and HDP or preeclampsia. Both univariate and multivariate logistic regression analyses were performed, adjusting for known risk factors for preeclampsia. P values below 0.05 were considered statistically significant. The statistical analysis was performed using SPSS version 26.0 (SPSS, Statistical Package for the Social Sciences, IBM, NY, USA) and RStudio (Version 1.0.136, Rstudio, Inc., Boston, MA, USA) statistical software.

## 3. Results

### 3.1. Study Population

The analysis included 1473 twin pregnancies, with 152 (10.3%) pregnancies complicated by HDP and 110 cases of preeclampsia. The comparison of the twin pregnancies according to whether they developed HDP or not is presented in Table 1. Compared with women with normotensive pregnancies, women in the HDP group were more likely to be nulliparous (67.1% vs. 55.2%, *p* = 0.005) and had significantly higher BMI (median (IQR) 25.7 (22.3–29.7) kg/m^2^ vs. 24.3kg/m^2^ (21.8–27.8), *p* = 0.009). When compared to normotensive twin pregnancies, those complicated by HDP delivered at a significantly earlier gestation (median (IQR) 36.1 (34.6–37.1) weeks vs. 36.9 (35.0–37.6) weeks, *p* < 0.001), were more likely to be admitted to the High Dependency Unit (HDU) or Intensive Care Unit (ICU) (19.1% vs. 4.4%, *p* < 0.001), and their babies more likely to be admitted to the neonatal unit (NNU) (38.8% vs. 30.7%, *p* = 0.040). They were also more likely to have both neonates small, whether as SGA (7.9% vs. 2.9%, *p* = 0.001) or FGR (3.3% vs. 0.5%, *p* < 0.001). A total of 176 cases of sFGR were identified: 33 (21.7%) in the HDP group and 143 (10.8%) in the control group (*p* < 0.001).

### 3.2. The Association of Intertwin Birthweight Discordance and FGR in Twin Pregnancies with HDP

The median (IQR) intertwin birthweight discordance was significantly greater in the HDP group when compared to the normotensive pregnancies (12.9%; 4.8–19.5 vs. 9.3%; 4.1-16.0, *p* = 0.003). Intertwin discordance ≥15% (44.7% vs. 28.3%, *p* < 0.001), ≥20% (25.0% vs. 17.3%, *p* = 0.019), ≥25% (15.8% vs. 8.1%, *p* = 0.002) were significantly more common in the HDP group compared to the normotensive group (Table 2). The results of the logistic regression analysis are shown in Table 2. The association between intertwin discordance and HDP remained significant even after adjusting for maternal age, BMI, parity, assisted conception and gestational age at delivery (Table 2).

The association between fetal growth disorders and HDP in twin pregnancies was stronger when twin standard birthweight charts were used rather than the singleton equivalent (Table 3). The prevalence of SGA of at least one twin (40.1% vs. 23.1%, *p* < 0.001; aOR = 2.34, 95% CI: 1.60–3.44, *p* < 0.001) and FGR of at least one twin (17.1% vs. 8.7%, *p* = 0.001; aOR = 2.17, 95% CI: 1.29-3.64, *p* = 0.004), defined by twin standard birthweight charts, were significantly higher in hypertensive than in normotensive pregnancies. The co-presence of SGA affecting at least one twin and intertwin discordance ≥25% was also significantly associated with HDP (aOR = 2.03, 95% CI: 1.16-3.53, *p* = 0.013).

The sensitivity analysis according to the chorionicity is shown in Table 4. These associations between intertwin discordance or FGR and HDP remained statistically significant for dichorionic but not for monochorionic twin pregnancies.

### 3.3. The Association of Intertwin Birthweight Discordance and FGR with HDP or Preeclampsia after Adjustment for the Known Risk Factors for Preeclampsia

Maternal age, BMI and nulliparity, SGA in at least one twin and intertwin birthweight discordance ≥25% were significantly associated with HDP in the univariate logistic regression (Table 5). However, only BMI (OR 1.06, 95% CI 1.02–1.09, *p* = 0.001), nulliparity (OR 1.81, 95% CI 1.23–2.68, *p* = 0.003) and SGA in at least one twin (OR 2.12, 95% CI 1.40–3.22, *p* < 0.001) remained significantly associated with HDP in the multivariate analysis. When the analysis was limited to pregnancies complicated by preeclampsia, SGA in at least one twin (OR 2.34, 95% CI 1.45–3.76, *p* < 0.001), BMI (OR 1.05, 95% CI 1.01–1.09, *p* = 0.007) and nulliparity (OR 1.74, 95 CI 1.10–2.74, *p* = 0.017) were associated with preeclampsia in the multivariate logistic regression analysis.

## 4. Discussion

### 4.1. Summary of Main Results

Our findings demonstrate that intertwin discordance ≥15%, ≥20%, ≥25%, SGA in at least one twin and FGR in at least one twin were significantly associated with HDP. This association was stronger when twin-specific reference birthweight charts were used compared to the use of singleton charts. The presence of SGA in at least one twin was an independent predictor of the risk of HDP or preeclampsia, even after adjusting for the known maternal risk factors for preeclampsia and the intertwin birthweight discordance.

### 4.2. Interpretation of Study Findings and Comparison with Published Literature

The association between fetal growth disorders and the development of hypertension in twin pregnancies has been investigated, with evidence of conflicting results [8,9,10,11,12,13]. In small retrospective studies, the association between SGA and preeclampsia was not detected. This was primarily because authors used singleton-based birthweight reference charts to define SGA, leading to a very high rate of SGA twins in both normotensive and hypertensive twin pregnancies [10,11]. Conversely, SGA was associated with an increased risk of preeclampsia in studies where a twin-specific birthweight chart was used [8,12,13]. In our study, SGA and FGR were associated with HDP using either singleton or twin charts; however, these associations were stronger when twin-specific charts were used. Little is known about the association between intertwin birthweight discordance and HDP. Qiao et al. recently showed that growth discordance, adjusted for SGA, is an independent risk factor for the occurrence of preeclampsia in twin pregnancies. However, the authors did not specify which reference was used to calculate birthweight centile [9].

Our results reveal that both intertwin birthweight discordance and either SGA or FGR complicating a twin pregnancy was associated with HDP or preeclampsia, but the presence of SGA in at least one twin seems to be the strongest risk factor. These data are consistent with previous studies that confirmed the association between fetal growth disorders and HDP in dichorionic twin pregnancies. We could not demonstrate a similar relationship in monochorionic twin pregnancies, which could be due to the different pathology of growth disorders (unequal placental sharing) or the small sample size.

It is well known that placental insufficiency, which can be manifested as HDP or SGA, is related to inadequate remodeling of spiral arteries and, subsequently, to a malperfusion of the placenta. However, there is increasing evidence that placentas from twin pregnancies complicated by preeclampsia, fetal growth disorders, or both, showed a lower prevalence of histological lesions than those from their singleton counterparts [23,24,25,26]. Moreover, compared with SGA singletons, SGA twins were less likely to have any placental pathology, including maternal vascular malperfusion pathology (aOR 0.24), fetal vascular malperfusion pathology (aOR 0.62), hypercoiled cord (aOR 0.45), and placental weight less than the 10th centile (aOR 0.13). However, SGA twins were more likely to have a marginal or velamentous cord insertion compared with SGA singletons (aOR 13.82) [24]. Thus, growth disorders and HDP in twins might be caused by maternal failure to supply the increased demand of two fetuses rather than merely placental insufficiency. One possible explanation of the difference between twin and singleton pregnancies in the pathophysiology of FGR and HDP might be the maternal inability to adapt to and meet the greater placental demand in a twin pregnancy. When the maternal cardiovascular system fails to adequately supply the uteroplacental unit, placental hypoperfusion might lead to fetal growth disorders and/or maternal hypertension [27]. Impaired maternal cardiovascular system reported in conditions such as obesity, chronic hypertension or diabetes can boost this mechanism as showed by a higher BMI in the HDP group compared to the controls in our cohort [28].

Previous studies demonstrated that chorionicity is not associated with HDP [2,29,30]. However, monochorionic twin pregnancies seem to be less affected by hypertensive complications compared to dichorionic ones [2]. This might be due to a lower gestational age at delivery in monochorionic twins [31] and to a lower circulating blood volume in monochorionic twin pregnancies (single fetal-placental unit) with a subsequent better cardiovascular adaptation to the pregnancy compared to dichorionic twins (double fetal-placental unit) [32]. However, more studies are necessary to elucidate the differences between the two groups and to explain the pathogenesis of HDP in each group.

### 4.3. Clinical and Research Implications

The use of twin charts could reduce the risk of unnecessary iatrogenic prematurity in twin pregnancies, as fewer pregnancies are labeled as complicated by FGR [33,34]. Moreover, routine assessment of fetal Dopplers in these pregnancies is likely to stratify their risk and antenatal management, in particular the ideal timing of birth.

The association between FGR/SGA and HDP in twins might facilitate early identification of women at increased risk of developing hypertension during pregnancy. These women are likely to benefit from closer blood pressure monitoring that can be performed by using different approaches and tools. For instance, the introduction of telemonitoring system in pregnancies has made the blood pressure monitoring more accessible and cost-saving [35,36].

### 4.4. Strengths and Limitations

The main limitation of this study is its retrospective design. We could not identify the timing of each event during pregnancy as we evaluated the association between growth disorders at birth and HDP. We excluded pregnancies complicated by stillbirth, which could potentially represent the most severe end of the spectrum. The lack of data on placental histology in all the pregnancies does not allow us to confirm chorionicity or to investigate the association between the placental pathological findings, HDP and small twins. Other limitations include the lack of a singleton group to compare the magnitude of this association and the absence of Doppler data in the analysis which help to differentiate fetuses which are truly growth restricted from those which were constitutionally small.

The main strength is the large sample size, robust diagnostic criteria for FGR/SGA, HDP and preeclampsia and the comparison of singleton- and twin-based birthweight charts to calculate the birthweight percentile.

## 5. Conclusions

This study highlights the association between fetal growth abnormalities and maternal hypertension in twin pregnancies. Prospective studies are needed to clarify whether closer monitoring of blood pressure in twin pregnancies with fetal growth abnormalities could facilitate earlier diagnosis and improve the monitoring and management of HDP, and maternal and perinatal outcomes.

## Figures and Tables

**Table 1:** Baseline characteristics of twin pregnancies complicated by hypertensive disorders of pregnancy (HDP) compared to normotensive twin pregnancies.

	Normotensive Pregnancies (n = 1321)	Pregnancies with HDP (n = 152)	*p* Value
Maternal age in years, median (IQR)	33 (30–36)	34 (31–37)	0.227
Maternal body mass index in kg/m^2^, median (IQR)	24.3 (21.8–27.8)	25.7 (22.3–29.7)	0.009
Maternal weight in kg, median (IQR)	66.1 (59.5–76.0)	69.5 (61.7–80.7)	0.004
Nulliparous, n (%)	729 (55.2)	102 (67.1)	0.005
Racial origin, n (%)			0.914
• Caucasian	733 (59.9)	93 (63.3)	
• Afro-Caribbean	195 (15.9)	21 (14.3)	
• Asian	148 (12.1)	15 (10.2)	
• Mixed	13 (1.1)	2 (1.4)	
• Others	134 (11)	16 (10.9)	
Smokers, n (%)	68 (5.3)	7 (4.9)	0.821
Assisted conception, n (%)	359 (27.4)	52 (34.2)	0.072
Chorionicity, n (%)			0.107
• Dichorionic	1048 (79.3)	129 (84.9)	
• Monochorionic	273 (20.7)	23 (15.1)	
Gestational age at delivery in weeks, median (IQR)	36.9 (35.0–37.6)	36.1 (34.6–37.1)	<0.001
Admission to HDU/ICU, n (%)	58 (4.4)	29 (19.1)	<0.001
Larger twin birthweight in grams, median (IQR)	2560 (2205–2850)	2482 (2058–2830)	0.106
Smaller twin birthweight in grams, median (IQR)	2300 (1930–2560)	2100 (1733–2455)	0.001
Larger twin birthweight centile, median (IQR)			
(a) Twin standard	54.1 (34.5–74.6)	51.0 (29.0–71.4)	0.1
(b) Singleton standard	31.4 (15.3–52.1)	29.7 (15.5–50.6)	0.734
Smaller twin birthweight centile, median (IQR)			
(a) Twin standard	27.2 (11.4–47.9)	8.3 (5.9–40.0)	<0.001
(b) Singleton standard	10.6 (3.2–23.9)	6.3 (1.8–19.2)	0.002
Admission to NNU, n (%)	405 (30.7)	59 (38.8%)	0.04
Larger twin admitted to NNU, n (%)	335 (25.7)	47 (30.9)	0.128
Smaller twin admitted to NNU, n (%)	358 (27.1)	55 (36.2)	0.018

Abbreviations: HDU: High Dependency Unit; ICU: Intensive Care Unit; NNU: Neonatal Unit.

**Table 2:** The association between intertwin birthweight discordance and HDP.

	Normotensive Pregnancies (n = 1321)	Pregnancies with HDP (n = 152)	Unadjusted OR (95% CI)	*p* Value	Adjusted OR (95% CI) *	*p* Value
Intertwin birthweight discordance ≥15%, n (%)	374 (28.3)	68 (44.7)	2.05 (1.46–2.88)	<0.001	2.57 (1.69–3.91)	<0.001
Intertwin birthweight discordance ≥20%, n (%)	228 (17.3)	38 (25.0)	1.60 (1.08–2.37)	0.020	1.77 (1.09–2.88)	0.020
Intertwin birthweight discordance ≥25%, n (%)	107 (8.1)	24 (15.8)	2.13 (1.32–3.43)	0.002	2.10 (1.26–3.49)	0.004

* Adjustment for body mass index, parity and gestational age at delivery. Abbreviations: OR: odds ratio; CI: confidence interval.

**Table 3:** The association between intertwin birthweight discordance, fetal growth restriction (FGR) and HDP.

	Normotensive Pregnancies (n = 1321)	Pregnancies with HDP (n = 152)	Unadjusted OR (95% CI)	*p* Value	Adjusted OR (95% CI) *	*p* Value
	Twin standards					
SGA in at least one twin, n (%)	305 (23.1)	61 (40.1)	2.23 (1.58–3.16)	<0.001	2.34 (1.60–3.44)	<0.001
FGR in at least one twin, n (%)	115 (8.7)	26 (17.1)	2.16 (1.36–3.44)	0.001	2.17 (1.29–3.64)	0.004
SGA in at least one twin plus Intertwin birthweight discordance ≥25%, n (%)	93 (7.0)	20 (13.2)	2.00 (1.19–3.35)	0.008	2.03 (1.16–3.53)	0.013
	Singleton standards					
SGA in at least one twin, n (%)	642(48.6)	94 (61.8)	1.71 (1.21–2.42)	0.002	1.89 (1.30–2.75)	0.001
FGR in at least one twin, n (%)	320 (24.2)	48 (31.6)	1.44 (1.00–2.08)	0.048	1.40 (0.94–2.07)	0.094
SGA in at least one twin plus Intertwin birthweight discordance ≥25%, n (%)	101 (7.6)	23 (15.1)	2.15 (1.32–3.51)	0.002	2.15 (1.28–3.61)	0.004

* Adjustment for body mass index, parity and gestational age at delivery. Abbreviations: SGA: small for gestational age; FGR: fetal growth restriction; OR: odds ratio; CI: confidence interval.

**Table 4:** The association between intertwin weight discordance, fetal growth restriction and hypertensive disorders of pregnancy, stratified by chorionicity.

	Normotensive Pregnancies (n = 1321)	Pregnancies with HDP (n = 152)	*p* Value	OR (95% CI)	*p* Value
	Dichorionic twin pregnancies				
Birthweight discordance ≥25%, n (%)	77 (7.3)	21 (16.3)	0.001	2.45 (1.45–4.13)	0.001
SGA in at least one twin, n (%)	243 (23.2)	52 (40.3)	<0.001	2.24 (1.53–3.27)	<0.001
FGR in at least one twin, n (%)	84 (8.0)	21 (16.3)	0.002	2.23 (1.33–3.75)	0.002
SGA in at least one twin plus Intertwin birthweight discordance ≥25%, n (%)	65 (6.2)	17 (13.2)	0.003	2.29 (1.3–4.05)	0.004
	Monochorionic twin pregnancies				
Birthweight discordance ≥25%, n (%)	30 (11.0)	3 (13.0)	0.730	1.21 (0.34–4.33)	0.764
SGA in at least one twin, n (%)	62 (22.7)	9 (39.1)	0.077	2.19 (0.90–5.29)	0.083
FGR in at least one twin, n (%)	31 (11.4)	5 (21.7)	0.143	2.17 (0.75–6.25)	0.152
SGA in at least one twin plus Intertwin birthweight discordance ≥25%, n (%)	28 (10.3)	3 (13.0)	0.720	1.31 (0.37–4.70)	0.676

Abbreviations: SGA: small for gestational age; FGR: fetal growth restriction; HDP: hypertensive disorders of pregnancy; OR: odds ratio; CI: confidence interval.

**Table 5:** Univariate and multivariate logistic regression analysis to identify which maternal and fetal factors are associated with hypertensive disorders of pregnancy and preeclampsia.

	Univariable Analysis		Multivariable Analysis	
	OR (95% CI)	*p* Value	OR (95% CI)	*p* Value
Hypertensive disorders of pregnancy (n = 152)				
Maternal age in years	1.03 (1.00–1.06)	0.045	1.03 (1.00–1.07)	0.035
Maternal body mass index in kg/m^2^	1.04 (1.01–1.08)	0.009	1.06 (1.02–1.09)	0.001
Assisted conception	1.39 (0.97–1.98)	0.073	---	---
Nulliparous	1.66 (1.16–2.36)	0.005	1.81 (1.23–2.68)	0.003
SGA in at least one twin (twin standards)	2.23 (1.58–3.16)	<0.001	2.12 (1.40–3.22)	<0.001
Intertwin birthweight discordance ≥25%	2.13 (1.32–3.43)	0.002	1.34 (0.76–2.36)	0.307
Preeclampsia (n = 110)				
Maternal age in years	1.01 (0.98–1.05)	0.467	--	--
Maternal body mass index in kg/m^2^	1.04 (1.00–1.08)	0.047	1.05 (1.01–1.09)	0.007
Assisted conception	1.39 (0.92–2.20)	0.113	--	--
Nulliparous	1.72 (1.14–2.61)	0.010	1.74 (1.10–2.74)	0.017
SGA in at least one twin (twin standards)	2.44 (1.64–3.64)	<0.001	2.34 (1.45–3.76)	<0.001
Intertwin weight discordance ≥25%	2.33 (1.37–3.96)	0.002	1.47 (0.79–2.72)	0.224

Abbreviations: OR: odds ratio; CI: confidence interval; SGA: small for gestational age.

## References

[1] Adashi E.Y., Gutman R. (2018). Delayed Childbearing as a Growing, Previously Unrecognized Contributor to the National Plural Birth Excess. Obstet. Gynecol..

[2] Francisco C., Wright D., Benkő Z., Syngelaki A., Nicolaides K.H. (2017). Hidden high rate of pre-eclampsia in twin compared with singleton pregnancy. Ultrasound Obstet. Gynecol..

[3] Kalafat E., Abiola A., Thilaganathan B., Bhide A., Khalil A. (2020). The Association between Hypertension in Pregnancy and Preterm Birth with Fetal Growth Restriction in Singleton and Twin Pregnancy: Use of Twin Versus Singleton Charts. J. Clin. Med..

[4] Fox N.S., Rebarber A., Klauser C.K., Roman A.S., Saltzman D.H. (2010). Intrauterine Growth Restriction in Twin Pregnancies: Incidence and Associated Risk Factors. Am. J. Perinatol..

[5] Miller J., Chauhan S.P., Abuhamad A.Z. (2012). Discordant twins: Diagnosis, evaluation and management. Am. J. Obstet. Gynecol..

[6] D’Antonio F., Odibo A.O., Prefumo F., Khalil A., Buca D., Flacco M.E., Liberati M., Manzoli L., Acharya G. (2018). Weight discordance and perinatal mortality in twin pregnancy: Systematic review and meta-analysis. Ultrasound Obstet. Gynecol..

[7] Burton G.J., Jauniaux E. (2018). Pathophysiology of placental-derived fetal growth restriction. Am. J. Obstet. Gynecol..

[8] Proctor L.K., Kfouri J., Hiersch L., Aviram A., Zaltz A., Kingdom J., Barrett J., Melamed N. (2019). Association between hypertensive disorders and fetal growth restriction in twin compared with singleton gestations. Am. J. Obstet. Gynecol..

[9] Qiao P., Zhao Y., Jiang X., Xu C., Yang Y., Bao Y., Xie H., Ying H. (2020). Impact of growth discordance in twins on preeclampsia based on chorionicity. Am. J. Obstet. Gynecol..

[10] Sparks T.N., Nakagawa S., Gonzalez J.M. (2016). Hypertension in dichorionic twin gestations: How is birthweight affected?*. J. Matern. Neonatal Med..

[11] Fox N.S., Saltzman D.H., Oppal S., Klauser C., Gupta S., Rebarber A. (2014). The relationship between preeclampsia and intrauterine growth restriction in twin pregnancies. Am. J. Obstet. Gynecol..

[12] Wu D., Huang L., He Z., Huang X., Fang Q., Luo Y. (2015). Preeclampsia in Twin Pregnancies: Association with Selective Intrauterine Growth Restriction. J. Matern. Neonatal Med..

[13] Ferrazzani S., Merola A., De Carolis S., Carducci B., Paradisi G., Caruso A. (2000). Birth weight in pre-eclamptic and normotensive twin pregnancies: An analysis of discordance and growth restriction. Hum. Reprod..

[14] Khalil A., Rodgers M., Baschat A., Bhide A., Gratacos E., Hecher K., Kilby M.D., Lewi L., Nicolaides K.H., Oepkes D. (2016). ISUOG Practice Guidelines: Role of ultrasound in twin pregnancy. Ultrasound Obstet. Gynecol..

[15] Sepulveda W., Sebire N.J., Odibo A., Psarra A., Nicolaides K.H. (1996). Prenatal determination of chorionicity in triplet pregnancy by ultrasonographic examination of the ipsilon zone. Obstet. Gynecol..

[16] Poon L.C., Tan M.Y., Yerlikaya G., Syngelaki A., Nicolaides K.H. (2016). Birth weight in live births and stillbirths. Ultrasound Obstet. Gynecol..

[17] Dias T., Ladd S., Mahsud-Dornan S., Bhide A., Papageorghiou A.T., Thilaganathan B. (2011). Systematic labeling of twin pregnancies on ultrasound. Ultrasound Obstet. Gynecol..

[18] Monaghan C., Kalafat E., Binder J., Thilaganathan B., Khalil A. (2019). Prediction of adverse pregnancy outcome in monochorionic diamniotic twin pregnancy complicated by selective fetal growth restriction. Ultrasound Obstet. Gynecol..

[19] Robinson H.P., Fleming J.E.E. (1975). A CRITICAL EVALUATION OF SONAR “CROWN-RUMP LENGTH” MEASUREMENTS. BJOG Int. J. Obstet. Gynaecol..

[20] Khalil A., Beune I.M., Hecher K., Wynia K., Ganzevoort W., Reed K., Lewi L., Oepkes D., Gratacos E., Thilaganathan B. (2019). Consensus definition and essential reporting parameters of selective fetal growth restriction in twin pregnancy: A Delphi procedure. Ultrasound Obstet. Gynecol..

[21] Ananth C.V. (1998). Standards of Birth Weight in Twin Gestations Stratified by Placental Chorionicity. Obstet. Gynecol..

[22] Tranquilli A., Dekker G., Magee L., Roberts J., Sibai B., Steyn W., Zeeman G., Brown M. (2014). The classification, diagnosis and management of the hypertensive disorders of pregnancy: A revised statement from the ISSHP. Pregnancy Hypertens..

[23] Aviram A., Giltvedt M.K., Sherman C., Kingdom J., Zaltz A., Barrett J., Melamed N. (2018). The role of placental malperfusion in the pathogenesis of preeclampsia in dichorionic twin and singleton pregnancies. Placenta.

[24] Kibel M., Kahn M., Sherman C., Kingdom J., Zaltz A., Barrett J., Melamed N. (2017). Placental abnormalities differ between small for gestational age fetuses in dichorionic twin and singleton pregnancies. Placenta.

[25] Matthews K.C., Fox N.S., Rebarber A. (2019). The Association between Placental Histopathology, Fetal Growth Restriction, and Preeclampsia in Twin Pregnancies. Am. J. Perinatol..

[26] Weiner E., Feldstein O., Schreiber L., Grinstein E., Barber E., Dekalo A., Bar J., Kovo M. (2017). Placental Component and Pregnancy Outcome in Singleton versus Twin Pregnancies Complicated by Preeclampsia. Fetal Diagn. Ther..

[27] Thilaganathan B., Kalafat E. (2019). Cardiovascular System in Preeclampsia and Beyond. Hypertension.

[28] Buddeberg B., Sharma R., O’Driscoll J.M., Agten A.K., Khalil A., Thilaganathan B. (2019). Cardiac maladaptation in obese pregnant women at term. Ultrasound Obstet. Gynecol..

[29] Carter E.B., Bishop K.C., Goetzinger K.R., Tuuli M.G., Cahill A.G. (2015). The impact of chorionicity on maternal pregnancy outcomes. Am. J. Obstet. Gynecol..

[30] Lucovnik M., Blickstein I., Lasič M., Fabjan-Vodušek V., Bržan-Simenc G., Verdenik I., Tul N. (2016). Hypertensive disorders during monozygotic and dizygotic twin gestations: A population-based study. Hypertens. Pregnancy.

[31] Wright D., Wright A., Nicolaides K.H. (2020). The competing risk approach for prediction of preeclampsia. Am. J. Obstet. Gynecol..

[32] Ghi T., Dall’Asta A., Franchi L., Fieni S., Gaibazzi N., Siniscalchi C., Pedrazzi G., Montaguti E., Degli Esposti D., Carpano M.G. (2018). The Effect of Chorionicity on Maternal Cardiac Adaptation to Uncomplicated Twin Pregnancy: A Prospective Longitudinal Study. Fetal Diagn. Ther..

[33] Kalafat E., Sebghati M., Thilaganathan B., Khalil A., Bahamie A., Bhide A., Deans A., Egbor M., Ellis C., Gandhi H. (2019). Predictive accuracy of Southwest Thames Obstetric Research Collaborative (STORK) chorionicity-specific twin growth charts for stillbirth: A validation study. Ultrasound Obstet. Gynecol..

[34] (2019). FIGO Working Group on Good Clinical Practice in Maternal-Fetal Medicine Good clinical practice advice: Role of ultrasound in the management of twin pregnancy. Int. J. Gynecol. Obstet..

[35] Khalil A., Perry H., Lanssens D., Gyselaers W. (2019). Telemonitoring for hypertensive disease in pregnancy. Expert Rev. Med. Devices.

[36] Xydopoulos G., Perry H., Sheehan E., Thilaganathan B., Fordham R., Khalil A. (2019). Home blood-pressure monitoring in a hypertensive pregnant population: Cost-minimization study. Ultrasound Obstet. Gynecol..

